# Lessons from the ‘Iressa’ Expanded Access Programme: gefitinib in special non-small-cell lung cancer patient populations

**DOI:** 10.1038/sj.bjc.6601479

**Published:** 2003-12-10

**Authors:** R Stahel, A Rossi, L Petruzelka, P Kosimidis, F de Braud, M M Bernardo, P-J Souquet, H Soto Parra, C Gridelli

**Affiliations:** 1 Klinik und Poliklinik für Onkologie, Universitätsspital, Rämistrasse 100, CH - 8091, Zurich, Switzerland; 2 SG Moscati Hospital, Avellino, Italy; 3 University Hospital, Charles University, Prague, Czech Republic; 4 An Tsoha and Vas Sofia Aven, Athens, Greece; 5 Istituto Europeo di Oncologia, Milan, Italy; 6 Hospital Sta Antonio Dos Capuchos, Lisbon, Portugal; 7 Centre Hospitalier Lyon-Sud, Pierre Benite, France; 8 Istituto Clinico Humanitas, Rozzano, Milan, Italy

**Keywords:** gefitinib (‘Iressa’, ZD1839), EGFR-TKI, NSCLC, elderly, performance status, chemonaive

## Abstract

Some subgroups of patients with advanced/metastatic non-small-cell lung cancer (NSCLC) are frequently considered ineligible for the aggressive, platinum-based combination chemotherapy that is the recommended treatment. Elderly patients may have a poorer tolerance of chemotherapy due to impaired organ function and frequent comorbidities; patients with poor performance status (PS; ⩾2 due to NSCLC and/or coexisting illnesses) are often considered unfit for chemotherapy; other patients may be unable or unwilling to endure the toxicity or inconvenience of chemotherapy. These patient groups may benefit from novel, relatively nontoxic treatment modalities. Gefitinib (‘Iressa’, ZD1839) 250 mg day^−1^ is well tolerated and has proven antitumour and symptom improvement activity in patients with previously treated NSCLC. Phase II trials (IDEAL 1 and 2) of gefitinib in advanced/metastatic NSCLC included 70 out of 425 (16.5%) patients with PS ⩾2, and their response rate, clinical benefit rate and rates of adverse events were similar to those of the overall trial population. In addition, many patients with advanced/metastatic NSCLC with poor PS or advanced age have received gefitinib 250 mg day^−1^ in an Expanded Access Programme (EAP). Observations from the EAP support those of IDEAL 1 and 2, and indicate that gefitinib 250 mg day^−1^ warrants further investigation in these patient groups.

Current standard combination chemotherapy regimens for advanced non-small-cell lung cancer (NSCLC) are effective in prolonging survival, preventing or reducing tumour-related symptoms and maintaining quality of life (Schiller, 2001). Given the equal effectiveness of current regimens, the selection of therapy for an individual patient is mainly based on the issue of side effects and the ease of administration in a given setting.

Many patients, including the elderly and unfit, cannot be considered eligible for combination chemotherapy in order to avoid undue toxicity. In Europe, >30% of patients with NSCLC are aged >70 years (Gridelli, 2002), and patients in this age group frequently experience a progressive decline in organ function. In addition, many patients with NSCLC have multiple comorbidities that impact on their performance status (PS), and other patients are too ill because of symptoms from advanced disease, such as weight loss, cough and fatigue (Govindan, 2003). Advanced age and poor PS are factors that need to be considered when choosing therapy.

Platinum-based combination chemotherapy can be associated with high rates of severe adverse effects, including haematological adverse events. Grade 4–5 events occurred in 57–70% of patients in a large study (*n*=1146) comparing four chemotherapy regimens (cisplatin+paclitaxel, cisplatin+gemcitabine, cisplatin+docetaxel and paclitaxel+carboplatin) in patients with advanced NSCLC (Schiller, 2001). In patients with NSCLC and a PS of 2 (*n*=64), a comparative study found grade 3–4 haematological toxicities in >50% of patients in each of four treatment groups (paclitaxel+cisplatin, cisplatin+gemcitabine, cisplatin+docetaxel and paclitaxel+carboplatin) (Sweeney *et al*, 2001). While it is generally accepted that haematological toxicity is largely a laboratory toxicity, grade 4 febrile neutropenia does occur in 3–14% of patients, depending on the regimen chosen (Schiller, 2001). Therefore, a proportion of elderly patients and patients with poor PS are often considered ineligible for aggressive, platinum-based combinations. In addition, second-line treatment options are limited, and only patients with good PS are considered candidates for second-line treatment.

Patients excluded or deterred from active therapy for advanced NSCLC may benefit from new medications that are easy to take, cause limited adverse events and have a low risk of drug interactions. Alternative strategies need to be explored with the aim of specifically tailoring therapy to the needs of these groups.

Novel, biologically targeted agents that aim to disrupt specific properties of a tumour's activities, while interfering with host functions to a relatively minor degree, promise a higher therapeutic margin and lower toxicity than traditional therapies. One such agent, the epidermal growth factor receptor tyrosine kinase inhibitor (EGFR-TKI) gefitinib (‘Iressa’, ZD1839) presents new possibilities for patient groups considered unfit for aggressive chemotherapy.

Gefitinib monotherapy in patients with advanced NSCLC has been investigated in two large, multicentre, randomised Phase II trials, ‘Iressa’ Dose Evaluation in Advanced Lung cancer (IDEAL) 1 and 2. The majority of patients in IDEAL 2 were receiving gefitinib as at least fourth-line therapy, while it was second- or third-line therapy for all of the patients in IDEAL 1. The objective tumour response rate at 250 mg day^−1^ in IDEAL 1 was 18.4%. In IDEAL 2, the more heavily pretreated trial population, the objective response rate was 11.8% for the 250 mg day^−1^ dose. Objective responses were seen irrespective of the number of prior chemotherapy regimens. In addition, gefitinib was found to improve NSCLC-specific symptoms at comparable rates in the two trials (40.3 and 43.1% of patients in IDEAL 1 and 2, respectively) (Douillard *et al*, 2002; Natale *et al*, 2002). Adverse events at the 250 mg day^−1^ dose were generally mild and reversible grade 1/2 (National Cancer Institute common toxicity criteria (CTC) version 2.0) diarrhoea and skin reactions (rash, pruritus, dry skin, acne), with a low incidence of grade 3/4 adverse drug reactions, dose reductions and withdrawals due to drug-related adverse events (Herbst and Kies, 2002; Kris *et al*, 2003). Gefitinib was not associated with haematological toxicity or stomatitis. Overall, the results showed that 250 mg day^−1^ gefitinib was as effective as 500 mg day^−1^, and the superior tolerability of 250 mg day^−1^ makes this the recommended dose (Fukuoka *et al*, 2003; Kris *et al*, 2003).

Recently, data became available from centres that have administered gefitinib 250 mg day^−1^ to patients with advanced NSCLC on a compassionate-use basis, as part of an Expanded Access Programme (EAP). The Iressa Clinical Experience (ICE) meeting of investigators held in Madrid, Spain (June 2003) allowed clinicians to share their experience of gefitinib in patient groups that included the elderly and unfit as well as patients unwilling to tolerate chemotherapy. Some of these patients received gefitinib 250 mg day^−1^ as first-line therapy. This paper brings together results from IDEAL 1 and 2 and from the EAP, and discusses treatment approaches for groups of patients with special therapy requirements.

## CURRENT APPROACHES TO SPECIAL PATIENT POPULATIONS WITH NSCLC, AND CLINICAL EXPERIENCE FROM THE GEFITINIB EAP

### Patients with poor PS (⩾2)

Studies have shown that PS at diagnosis is a powerful prognostic indicator in patients with NSCLC. For several types of treatment, poor PS has been associated with reduced survival, poor response rate or increased toxicity. In a study involving patients with NSCLC treated with docetaxel, patients with PS 0–1 had a median overall survival of 11.3 months compared with 3.8 months for patients with PS 2 (*P*<0.0001) (Kosmidis *et al*, 1997). A further study, comparing combination chemotherapy (paclitaxel+carboplatin *vs* paclitaxel+gemcitabine), found that patients with PS 2 had a lower response rate (11%) than patients with PS 0–1 (34%) (*P*<0.0001) (Kosmidis *et al*, 2002). In ECOG study E1594, comparing paclitaxel and cisplatin with three other chemotherapy doublets in patients with advanced NSCLC, patients with PS 2 experienced a large number of adverse reactions and overall poor survival. In the course of the trial, accrual of patients with PS 2 was discontinued due to a perceived rate of unacceptable toxicity that was judged to be related to the disease process rather than treatment (Sweeney *et al*, 2001). Taken together, the results of these studies support the view that patients with poor PS have special treatment requirements.

Based on current data and treatment options, a recent European consensus panel recommended single-agent chemotherapy (e.g. gemcitabine, vinorelbine, taxanes) as the preferred first-line treatment for patients with advanced NSCLC and PS ⩾2, with carboplatin-based doublets or attenuated-dose cisplatin-based doublets as alternatives).

#### Experience with gefitinib

Phase II trials (IDEAL 1 and 2) of gefitinib in advanced/metastatic NSCLC included 70/425 (16.5%) patients with PS ⩾2. The majority (43 out of 70) of these were in IDEAL 2, where they formed 19.9% of the overall population. The efficacy of gefitinib in patients with PS ⩾2 in IDEAL 2 was comparable with its efficacy in IDEAL 2 overall: patients with PS 2–3 had a response rate of 14% and a clinical benefit rate of ∼40% compared with rates in the whole trial population of 11.8 and 42.2%, respectively.

At the SG Moscati Hospital, Avellino, Italy, 41 patients with PS ⩾2 have been treated with gefitinib 250 mg day^−1^ as part of the gefitinib EAP (Gridelli *et al*, 2003; Gridelli [a], ICE abs). (See appendix for ICE abstracts.) Patient characteristics are shown in Table 1

**Table 1:** Demographics of a group of patients with NSCLC and poor PS treated with gefitinib 250 mg day^−1^ in an EAP (Maione, ICE abs; Gridelli *et al*, 2003). Reproduced with permission.

Patients, *n*	41
Male : female, *n*	26 : 15
Median age (range) (years)	60 (38–69)
*Disease stage*, n	
IIIb	4
IV	37
Eastern Cooperative Oncology Group	
performance status 2/3, *n*	29/12
*Tumour histology*, n	
Squamous-cell carcinoma	17
Adenocarcinoma	21
Undefined	3
*Prior chemotherapy regimens,* n	
0/1/⩾2	1/16/24

. From 39 evaluable patients, two patients with adenocarcinoma reported a partial response (PR) and five patients had stable disease (SD), giving a disease control rate of 17.1%. The most frequent adverse events were grade 1 diarrhoea in two patients and grade 2 elevation of liver transaminases in one patient. One female patient with brain metastases had a complete response (CR) in the brain (Maione, ICE abs). This patient had received two prior chemotherapy regimens and radiotherapy, and had a PS of 3 prior to gefitinib treatment. After beginning to take gefitinib (250 mg day^−1^) the patient became stable and after 2 months, her PS had improved to 1; CR in the brain was documented after 3 months. The patient continued to be stable at the time of reporting (April 2003), and had experienced no adverse events.

In all, 25 abstracts presented at this EAP investigators' meeting discussed >75 patients with poor PS. Clinical benefit in the form of objective response, stable disease or improved PS was described in many cases, with generally good tolerability. Notably, a complete response was seen in a 39-year-old patient, with no history of smoking, who had undergone three prior cycles of chemotherapy and had a PS of 2. The patient's PS returned to 0 after 8 weeks of gefitinib 250 mg day^−1^, and the patient remained on gefitinib therapy for 52 weeks, experiencing a mild treatment-related acneiform rash at week 2 that had decreased by week 7 (Gelibter, ICE abs). In another report, a patient with bronchioalveolar cell carcinoma and a PS of 2–3 experienced a PR lasting for 9 months, during which time the PS returned to 1. Adverse events comprised mild diarrhoea and intermittent skin rash (van Zandwijk [a], ICE abs). A further report detailed a patient with PS 4 who experienced a 2-month period of disease and symptom control while taking gefitinib, with no side effects (Vincent, ICE abs).

In addition to these reports, an interim analysis of EAP patients with PS 2–3 in the USA has shown a response rate of 13% and a clinical benefit rate of ∼30% (Wolf *et al*, 2003). In summary, gefitinib treatment was feasible and well tolerated in patients with PS ⩾2 treated in the EAP, producing objective responses and disease control rates that were comparable to those seen in IDEAL 1 and 2. Durable responses were seen in heavily pretreated patients with poor PS.

### Elderly patients

For elderly patients with advanced NSCLC, single-agent chemotherapy remains the standard treatment approach (Gridelli, 2002). However, many elderly patients with good PS are considered unsuitable for chemotherapy because of reduced organ function. In IDEAL 1 and 2, patients taking gefitinib had ages that ranged up to 85 and 84 years, respectively, and elderly patients formed a considerable proportion of the patients that were evaluated for efficacy and tolerability. Although no analyses have been carried out on these patients specifically, relevant data have been forthcoming from reports presented at the ICE meeting, including two reports that together detail 39 patients (Table 2

**Table 2:** Demographics of two groups of elderly patients with NSCLC treated with gefitinib 250 mg day^−1^ given in an EAP (Gridelli *et al*, 2003). Rerpoduced with permission.

**Investigators**	**Gridelli *et al* (2003)**	**Soto Parra *et al* (2003)**
Patients, *n*	18	41
Male : female, *n*	17 : 1	26 : 15
Median age (range) (years)	73.5 (70–80)	60 (38–69)
		
*Disease stage*, n		4
IIIb	3	37
IV	15	
Eastern Cooperative Oncology Group performance status 1/2/3, *n*	4/11/3	All ⩾1
		
*Tumour histology*, n		
Squamous-cell carcinoma	10	8
Adenocarcinoma	6	12
Bronchioalveolar	1	0
Undefined	1	11
		
Prior chemotherapy regimens, *n*		
0/1/⩾2	1/7/10	11/16/4

) (Gridelli *et al*, 2003; Gridelli [a], ICE abs; Soto Parra *et al*, 2003; Soto Parra [b], ICE abs). Disease control was seen in both studies (SD in two patients and 16 patients, respectively). In one of the studies (Soto Parra *et al*, 2003), the median survival was 4 months and 1-year survival was 26%. Disease control was seen in >50% of elderly patients, and patients with disease control tended to have improved median survival (Figure 1

**Figure 1:**
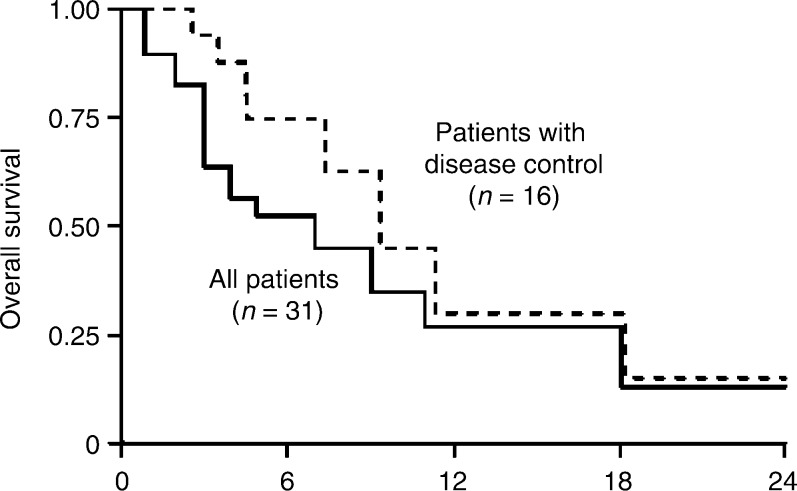
Kaplan–Meier plot of overall survival in a group of 31 elderly patients (mean (range) age 74 (70–82) years) with NSCLC taking gefitinib 250 mg day^−1^ as part of the gefitinib EAP at a centre in Rozzano, Italy (Soto Parra *et al*, 2003).

). The most common adverse events were skin rash (grade 1–2 in 42%; grade 3 in 10% of patients) and diarrhoea (grade 1–2 in 29%; grade 3 in 3% of patients).

Overall, gefitinib 250 mg day^−1^ showed reasonable efficacy and mild toxicity in these studies, and appears to be a valuable treatment option for elderly patients. Many other case reports from the EAP supported these findings.

### Chemonaive patients

Many patients with advanced NSCLC, including the elderly or unfit, are unable or unwilling to tolerate chemotherapy. Several cases of chemonaive patients treated with gefitinib 250 mg day^−1^ as part of the EAP were discussed by investigators.

**Figure 2:**
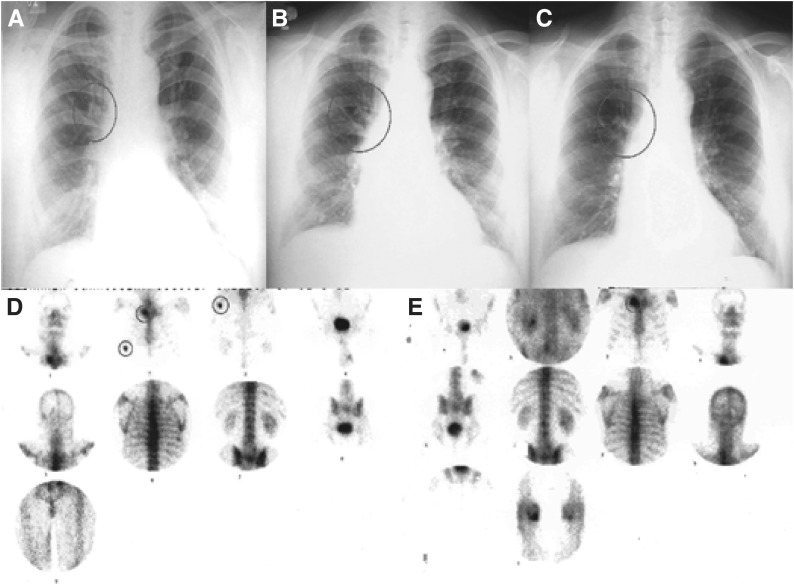
X-ray (**A–C**) and scintigraphy scans (**D**, **E**) showing primary and secondary tumour regression in a 64-year-old patient with NSCLC treated with first-line gefitinib 250 mg day^−1^. At diagnosis in March 2002 (**A**) and immediately prior to beginning gefitinib (**B**; July 2002), the patient had a primary tumour in the right upper lobe that partially responded (**C**; April 2003) for a sustained period, totalling 11 months at the time of reporting. Regression of bone metastases was evident 6 months after beginning gefitinib (**D**; March 2002, **E**; January 2003).

Petruzelka *et al* (ICE abs [b]) described a patient who chose not to receive chemotherapy as first-line treatment: a 64-year-old male exsmoker who presented with palpably enlarged lymph nodes in the right supraclavicular area. The supraclavicular mass was removed by nonradical surgery, and epidermoid carcinoma was confirmed by histopathology. Computed tomography scanning and X-ray imaging diagnosed a primary tumour in the right upper lobe (February 2002), and multiple metastatic bone lesions were also revealed. The patient was asymptomatic, but had several coexisting conditions including hypertension, obesity, diabetes mellitus, dyslipoproteinaemia and chronic obstructive pulmonary disease. He refused standard chemotherapy and received radiotherapy to the supraclavicular area (April-May 2002); he began to take gefitinib 250 mg day^−1^ in July 2002. In August 2002, he had a partial response (Figure 2

), confirmed 2 months later, and after a further 3 months he had regression of bone metastases (Figure 2) and no rib lesions on scintigraphy. His PR lasted >11 months, and was ongoing at the time of reporting. Improved quality of life was seen 2 months after starting gefitinib, which was generally well tolerated (grade 1 skin rash and grade 1/2 conjunctivitis).

Pesek and Eliasova described the case of a 70-year-old female nonsmoker who presented with stage IV pulmonary adenocarcinoma, with metastases to both lungs, mediastinal lymph nodes and vertebral column, and left-sided malignant pleural effusion (Pesek and Eliasova, ICE abs). She had severe comorbidity including cough, chest pain and fever, and a Karnofsky PS of 70%. She was offered standard first-line combination chemotherapy, but refused due to anxiety. She began gefitinib 250 mg day^−1^ 2 months later. Symptoms (cough, dyspnoea and chest pain) improved within 2 weeks of starting gefitinib, and the patient's quality of life improved. Nausea and diarrhoea were successfully managed, and the patient recorded a best response of SD and remission of pleural effusion. On tumour progression, the patient stopped gefitinib and began combination chemotherapy with gemcitabine, paclitaxel and carboplatin. A PR ensued, together with symptom improvement. At the time of reporting, 30 months after diagnosis, the patient was alive with slow, progressive, malignant disease.

These case reports show that gefitinib monotherapy can be efficacious in chemonaive patients, and can cause sustained responses at primary and metastatic sites, a finding that was supported by other EAP investigators' reports. In addition, it is clear that chemotherapy can be effective as second-line therapy following gefitinib.

## DISCUSSION

Data from the EAP show that, in elderly, unfit or chemonaive patients, the tolerability profile of gefitinib appears to be similar to that seen in the IDEAL trials and the general EAP population, supporting further investigation of gefitinib in these patient groups. The response rate with gefitinib in patients with PS 2–3 was 14% in IDEAL 2 and 13% in an interim analysis of the EAP. Despite the inclusion of many elderly (up to 85 years) patients in IDEAL 1 and 2, and inclusion of many patients with PS ⩾2 (12.9% in IDEAL 1 and 19.9% in IDEAL 2), 1-year survival was approximately 30%. This compares favourably with data from the IDEAL and EAP populations as a whole, and with data from a previous study for a population with similar baseline pretreatment characteristics: following initial regimens of a platin and docetaxel, patients (*n*=43) with a mean age of 51 (range 20 – 80) years went on to undergo subsequent lines of chemotherapy as required. Although >95% of patients had PS 0–1, 1–year survival was approximately 5%, and median survival was 4 months (Massarelli *et al*, 2002).

Case reports and case cohorts show that several elderly or unfit patients have experienced significant clinical benefit. However, defining the most appropriate patient groups for first-line gefitinib treatment raises questions that have yet to be answered. Ideally, clinical trials are needed to quantify benefits and inform treatment choices. Several ongoing clinical trials are investigating gefitinib in special patient populations in NSCLC. These include (i) an open Phase II study of gefitinib plus best supportive care in chemonaive patients considered ineligible for chemotherapy with advanced NSCLC; (ii) a Phase II trial of single-agent gefitinib in poor-PS patients with previously untreated advanced NSCLC; (iii) a Phase I trial of gefitinib tolerability in combination with irradiation with or without cisplatin in patients with inoperable stage III NSCLC; and (iv) a multicentre, randomised Phase II study of gefitinib and gemcitabine *vs* gefitinib and vinorelbine as first-line therapy for elderly patients with advanced NSCLC. Prior to the design of future trials, a retrospective analysis of current data could be informative, to assess the reasons for administering gefitinib in chemonaive patients and to allow stratification of patients according to PS and comorbidities.

Taken together, the data from IDEAL and the EAP suggest several important benefits for patient groups with special needs, and present a compelling case for further investigations of gefitinib in these groups. It is hoped that data will continue to emerge from the EAP to complement the findings of clinical trials, as well as informing the planning of future trials that will accurately define the scope of gefitinib monotherapy in NSCLC patient subgroups.
